# Two synchronous primary mesenteric neuroendocrine tumors in a patient: a case report

**DOI:** 10.3389/fsurg.2024.1323468

**Published:** 2024-04-04

**Authors:** Elissavet Symeonidou, Ariadni Fouza, Ioannis Gkoutziotis, Christina Nikolaidou, Panagiotis Petras, Konstantinos Mpallas

**Affiliations:** ^1^ 5th Surgical Department, Aristotle University of Thessaloniki, General Hospital Ippokrateio, Thessaloniki, Greece; ^2^ Department of Pathology, General Hospital Ippokrateio, Thessaloniki, Greece

**Keywords:** primary mesenteric neuroendocrine tumor, DOTATATE, laparoscopy, NET, bowel resection, synchronous primary mesenteric NET

## Abstract

Primary mesenteric neuroendocrine tumors represent a rare clinical entity, challenging to manage, while a combination of imaging is demanded in order to differentiate it from metastatic disease, and set the diagnosis. If the tumor is resectable, surgery is the fundament of the therapeutic approach. The appearance of a second primary mesenteric tumor suggests an unacquainted scenario. The current article presents a case of a 40-year-old woman, who underwent laparoscopic excision of a mesenteric tumor located close to the left pararenal space. Pathology with immunohistochemistry examination reported neuroendocrine tumor grade 2. No further treatment was necessary. Seven months later, 68-Gallium DOTATATE detected another primary neuroendocrine tumor located at the right retroperitoneal space without other lesions. The second tumor was also resected laparoscopically, and the pathology confirmed the diagnosis of another neuroendocrine tumor G2. The postoperative course was uneventful, and six months later the patient is disease-free. The adequacy of 68-Gallium DOTATATE for the preoperative diagnosis of primary mesenteric tumors, the role of the laparoscopic approach, and the extent of lymph node resection are matters addressed in this article.

## Introduction

Neuroendocrine tumors usually originate from the gastrointestinal tract (GIT) or the pancreas. Their presentation in the mesentery is rare, especially without identifying another primary lesion that would suggest metastatic potential. Even more uncommon is the presence of two synchronous primary mesenteric neuroendocrine tumors in a patient. In this article, we present a case of a 40-year-old woman who underwent laparoscopic excision of a mesenteric neuroendocrine tumor located close to the left pararenal space, and seven months later she was diagnosed with another neuroendocrine tumor located at the right retroperitoneal space. Upon admission, the patient complained of atypical abdominal pain and constipation, whereas nothing remarkable was detected either from clinical examination or from blood samples. Thorough screening was performed, including Computed Tomography (CT) and Magnetic Resonance Imaging (MRI). Further diagnostic evaluation with GIT endoscopy, Positron Emission Tomography (PET) scan, and 68-gallium DOTATATE, did not identify any other lesions. The laparoscopic approach was used in both operations uneventfully since no surrounding structures’ invasion was noticed. Pathology and immunohistochemistry confirmed the diagnosis both times. The current article addresses a rare clinical entity, underlines the importance of thorough diagnostic evaluation to exclude the possibility of metastasis, and suggests a laparoscopic approach for the surgical management of these cases.

## Case presentation

A 40-year-old Caucasian female presented to the outpatient department complaining of constipation and atypical abdominal pain. Clinical examination revealed a small palpable mass in the left upper quadrant, without signs of abdominal tenderness. Her medical history included hyperthyroidism, regulated with carbimazole. She reported neither allergies, nor previous surgeries, and she was a non-smoker. There was nothing remarkable regarding laboratory exams, family, and psychosocial history.

**Figure 1:**
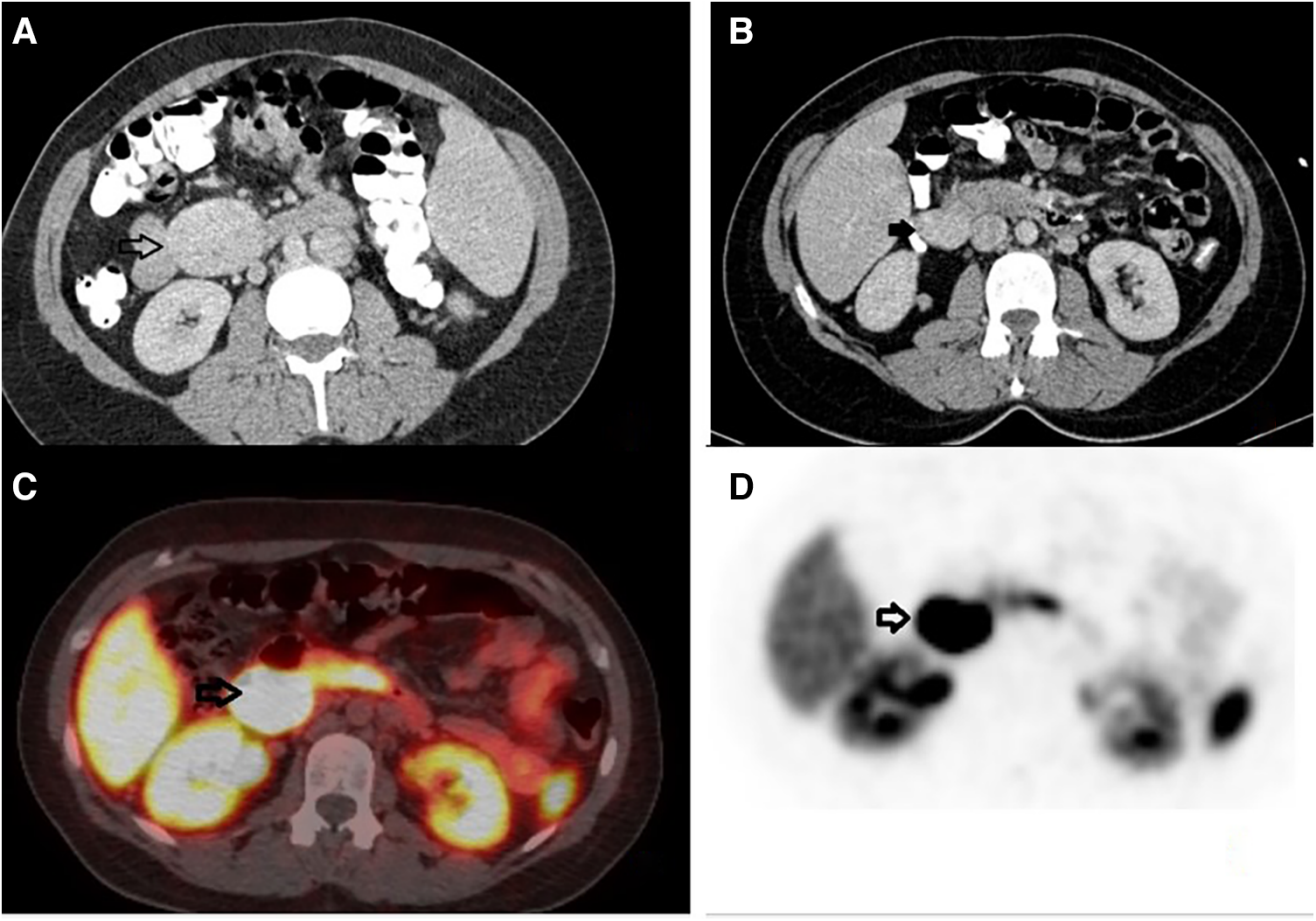
Preoperative imaging. (**A**) Computed tomography with intravenous contrast, transverse view, black arrow: an enhancing retroperitoneal mass, 4.1 cm in diameter, with clear boundaries, located in the left anterior pararenal space. (**B**) Computed tomography with intravenous contrast, transverse view, black arrow: an enhancing mass, 3.5 cm in diameter, located in the right retroperitoneal space. (**C**, **D**) Gallium-68 DOTATATE, transverse view, black arrows: a retroperitoneal mass, 3.5 cm in diameter, located medially to the upper pole of the right kidney, with increased uptake of the radioactive agent (SUV_max_ = 61), indicative of a neuroendocrine tumor.

A CT Scan revealed an enhancing retroperitoneal mass, 4.1 cm in diameter, with clear boundaries, located in the left anterior pararenal space, as illustrated in Figure 1A. MRI did not add further information. The differential diagnosis included paraganglioma, angioma, sarcoma, neurogenic tumor, and lymphoproliferative disorder, without being able to exclude other retroperitoneal tumors. The lesion was resectable and a decision to proceed with surgical excision was made, rather than ordering further imaging. Diagnostic laparoscopy was performed, followed by resection of the tumor found to be located in the jejunal mesentery. Neither organ mobilization nor bowel resection were needed. Pathology reported a low-grade neuroendocrine tumor (NET) of the gastrointestinal tract, grade 2 according to WHO/2019. Immunohistochemistry testing demonstrated positivity for synaptophysin, CD56, keratins AE1/AE3, and CDX2, as well as focal staining for chromogranin A, CK7, and CK20, and negativity for TTF-1, with a ki-67 index of 15%, findings consistent with NET. The multidisciplinary team (MDT) decided on observation.

**Figure 2:**
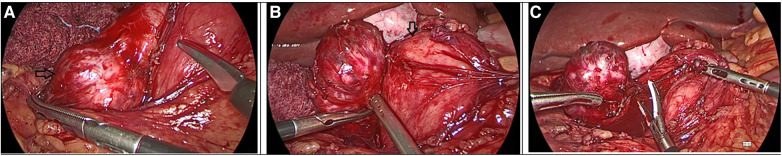
Intraoperative images from the second laparoscopy. (**A**) Black arrow: the mesenteric tumor arising from the ileac mesentery in close proximity to the duodenum. (**B**) Black arrow: the pancreatic head. (**C**) Completion of the laparoscopic excision of the tumor.

**Figure 3:**
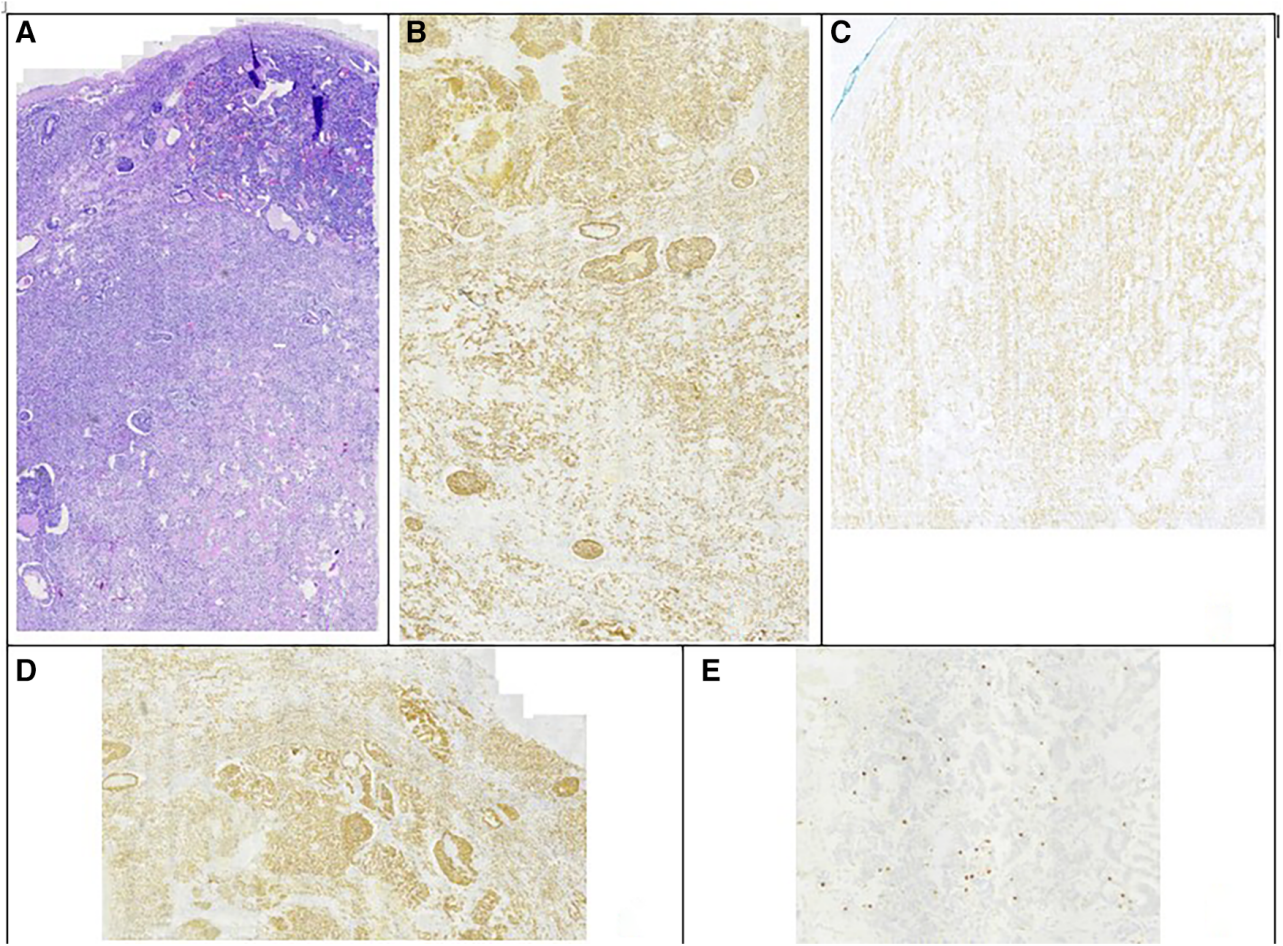
Immunohistochemistry testing of the second tumor. (**A**) Hematoxylin eosin staining. (**B**) Chromogranin A staining positive. (**C**) CD56 staining positive. (**D**) Synaptophysin staining positive. (**E**) ki-67 index 10%.

In the follow-up seven months later, CT reported complete resection of the mass of the left retroperitoneal space, without signs of residual or recurrent tumor. However, another mass that was not described earlier appeared; 3.5 cm in diameter, located in the right retroperitoneal space (Figure 1B). 18F-fluoro-D-glucose (FDG) PET followed, which revealed an enlargement close to the pancreaticoduodenal groove with mild enhancement, with a Standardized Uptake Value (SUVmax) 3.3, suggesting an enlarged lymph node. Upper GI endoscopy and colonoscopy were without any pathology. A PET-CT scan with Gallium-68 DOTATATE (tet-raazacyclododecanetetraacetic acid– DPhe1- Tyr3- octreotate) was performed afterward, reporting a retroperitoneal mass, 3.5 cm in diameter, located medially to the upper pole of the right kidney, close to inferior vena cava, with increased uptake of the radioactive agent (SUVmax = 61), indicative of a neuroendocrine tumor (Figures 1C,D). The patient was led to the operation theatre, where the tumor was identified in the ileac mesentery, as shown in Figures 2A,B, and entirely resected by laparoscopy (Figure 2C). After mobilization of the right hepatic flexure, an extended Kocher maneuver was performed, the tumor was dissected from the inferior vena cava and the Gerota's fascia, and removed from the abdominal cavity with a retrieval bag. No adhesions were encountered thanks to the previous laparoscopic approach. No enterectomy was demanded, and a drain was placed close to the pancreatic head. The postoperative period was uneventful and the patient was discharged on the 3rd postoperative day. The pathology report confirmed the diagnosis of a well-differentiated neuroendocrine tumor grade 2, according to WHO 2019/TNM 8th edition, with a ki-67 index of 10%, without any vascular or nerve infiltrations. Immunohistochemistry testing was positive for synaptophysin, chromogranin A, CKAE1/AE3, CD56, and ISLET-1, whereas it was negative for TTF-1, CK7, CK20, CDX2 and somatostatin (Figure 3). Based on the pathology results, there are some differences between the two tumors. The patient did not receive any further therapy, as the tumor was Grade 2, and completely resected. After 9 months of follow-up, the patient is disease-free. In particular, serum chromogranin A levels measured every three months are within normal range (<100 μg/L), as well as 24-hour urinary 5-hydroxyindoleacetic acid (5-HIAA). A PET-DOTATATE scan that was performed 6 months after the second operation showed no signs of neuroendocrine tumor.

**Figure 4:**
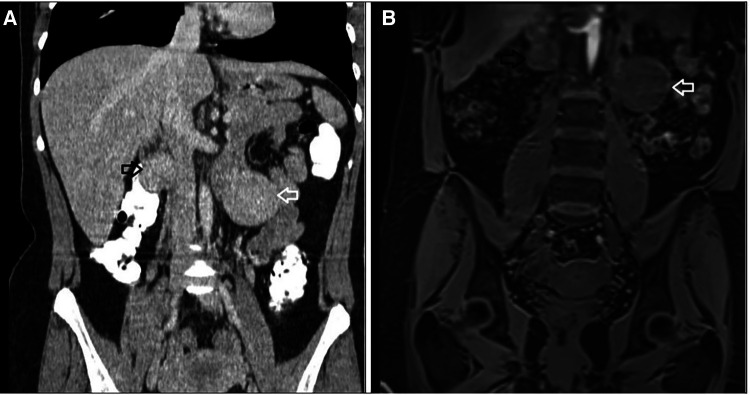
Imaging before the first surgery where both tumors are apparent. (**A**) Computed Tomography with intravenous contrast, coronal view, (**B**) MRI, coronal view: white arrow indicating the 4.1 cm retroperitoneal mass located in the left anterior pararenal space, black arrow indicating the tumor located in the right retroperitoneal space.

The reappearance of a second primary mesenteric tumor was surprising. Looking again carefully at the imaging before the first operation (Figure 4), it appeared that the second lesion was visible, although smaller in size, but it was not described either in the CT or in the MRI report. This leads us to the conclusion that our patient had two synchronous primary mesenteric neuroendocrine tumors. Even though the second lesion increased in size after seven months, still, no other lesion from the small intestine or the pancreas was noted in the follow-up imaging with DOTATATE.

The patient appears very satisfied with her postoperative course of treatment and she is very cooperative with her follow-up appointments, which include blood tests, serum chromogranin, 24-hour urinary 5-HIAA levels every 3 months, and a CT scan 12 months after surgery.

## Discussion

Neuroendocrine neoplasms (NENs) are divided into well-differentiated neuroendocrine tumors (NETs) and poorly- differentiated neuroendocrine carcinomas (NECs). Further classification, according to the World Health Organisation (WHO) 2022, is based on the ki-67 index, which is a prognostic marker, and divides NENs further into NET G1, G2, G3, and NEC G3. Their prevalence and incidence have been increasing worldwide over the years (1).

Regarding etiology, NENs originate from the enterochromaffin cells, otherwise called Kulchitsky cells, which are neuroendocrine cells located in the epithelium of the gastrointestinal tract, and play a significant role in GI motility and secretion (2). For this reason, the majority of NETs are located in the GIT (90% of them appearing in the appendix, the small intestine, and the rectum) (2), or the pancreas, while extra-GIT primary NENs are infrequent. If an extra-GIT NEN is diagnosed, thorough imaging should be applied, in order to rule out the possibility of it being metastasis, which has been reported in 40%–80% of GIT-NETs (2), and to identify the primary tumor (2).

Primary tumors of the mesentery, include usually benign conditions such as fibromas, neurofibromas, Schwannomas, paragangiomas, lipomas, teratomas, germ cell tumors, Castleman disease, sclerosing mesenteritis, and other mesenchymatic tumors composed of smooth muscle cells, blood vessels, or fat, as less frequently NETs and sarcomas (1–3).

Besides their common location in ileac or jejunal mesentery, primary mesenteric NETs have been reported to be located in the gastrohepatic ligament (3), and even in the mesocolon (4). They can be sporadic, or associated with a syndrome, such as multiple endocrine neoplasia type 1, neurofibromatosis type 1, and von Hippel-Lindau (5).

Preoperative diagnosis of mesenteric NETs is challenging. Multiphase CT or Magnetic Resonance Imaging (MRI) remains the first approach, but it cannot always detect mesenteric or intestine NETs of small size. Somatostatin-receptor- based imaging (SSTR) is sensitive for the diagnosis of NETs, however, it can miss tumors less than 2 cm (6). 68-Gallium DOTATATE PET/CT has higher sensitivity in detecting unknown primary NET tumors and possible metastatic lesions. In combination with the small amount of radiation exposure, it is an essential tool for the evaluation of mesenteric NETs. In a prospective study with 131 participants with gastro-entero-pancreatic NETs and unknown primary sites (7), 95.1% of the lesions were detected with 68-Ga DOTATATE PET, in comparison with conventional anatomic imaging (CT, MRI) which detected 45.3% of the lesions and SPECT/CT, which detected 30.9% respectively, whereas only in 4 cases all available imaging was negative. In the same study, the additional information 68-Ga DOTATATE PET provided, changed the course of treatment in 32.8% of the patients, proving the significant impact of this examination in medical and surgical management of NETs. According to a metaanalysis including 22 studies and 2015 participants, 68-Ga DOTATATE PET had sensitivity of 93% and specificity of 91% (8). In addition, it is a useful tool for the evaluation of possible recurrence (9), and in combination with FDG PET/CT was found to be helpful for the therapeutic approach of GEP-NETs, especially of the poorly- differentiated ones (9). However, there is a possibility that small intestinal NETs with a diameter less than 5 mm might not be apparent even in 68-Gallium PET (5). In our case, 68-Ga DOTATATE PET was used before the second operation and after the histologic confirmation of the first mesenteric NET, in particular six months after the first operation, excluding other possible primary lesions.

Surgical resection is the fundament of the therapeutic approach for patients with resectable disease. Careful palpation of the entire small intestine during laparotomy is recommended by some authors (10) in addition to imaging. In our case, because both surgeries were performed entirely laparoscopic, no palpation was applicable. However careful inspection of the small bowel and the liver was performed and no other pathology was found. Only a few cases with laparoscopic resection of mesenteric NET, followed by careful evaluation of the whole abdominal cavity and especially the small intestine and the liver, are reported in the literature (3, 11, 12), suggesting that the laparoscopic approach is a feasible option for the management of primary mesenteric NETs. Yamanuha et al. (12) combined the laparoscopic resection with lymph node biopsies. Lymph node metastases from a primary mesenteric tumor have been described, and they are associated with worse prognosis (13).

However, the necessity and the extent of the lymph node resection is a controversial topic. According to a recent review by Hallet and Law (14) regarding the small intestine NETs G1 and G2, for clinically negative lymph nodes, a resection of a minimum number of 8 was necessary for staging, despite the fact that no survival benefit was proven. For clinically positive lymph node involvement, a four stage lymphadenectomy is proposed (locoregional, in the origin of SMA, along the SMA, and in the retroperitoneal space behind the pancreatic head), ideally mesentery-sparing, with precaution to preserve intestinal length and function. In a retrospective study, Bartsch et al. (15) proposes a retrograde vessel-sparing lymphadenectomy for small intestine NETs. For primary mesenteric NETs, there are not enough data in order to establish clear recommendations, although many authors have proceeded with lymph node sampling. Subrenal retrocaval and interaortocaval lymph node resection has also been reported in the literature, for clinically positive lymph nodes (5).

According to Morishita et al. (1), by 2022 only 11 cases of primary mesenteric NETs are reported in the literature. Usually they are non-functional tumors and mainly asymptomatic (16). However, Shogbesan et al. (17) reported a case of primary mesenteric NET presenting with carcinoid syndrome, with symptoms including rashes and diarrhea. Liver metastases have also been described (18, 19), as well as ectopic Cushing's syndrome (20).

Only one similar case with two synchronous primary mesenteric neuroendocrine tumors was found in the literature. Kamath et al. published a case of synchronous primary mesenteric carcinoid tumors in a 38-year-old man (16).

Last but not least, follow-up should include 68-Ga DOTATATE PET scan, in order to exclude the possibility of a missed primary NET, diagnose tumor progression, lymph node involvement, distant metastasis, and therefore predict the prognosis (21). 68-Ga DOTATATE PET has proven a curve of 0.98 for identifying NET, with sensitivity and specificity of 93% and 96% respectively (21), suggesting an essential tool for the diagnosis and follow-up of these cases.

## Conclusion

Primary mesenteric NETs suggest a very rare clinical entity, with only a few reports found in the literature. Only one case with two primary mesenteric NETs appearing in the same patient has been reported in the literature. Thorough diagnostic evaluation in order to rule out the possibility of metastases and the identification of another primary tumor is crucial, and has been performed in this case. The use of 68-Ga-DOTATATE PET CT imaging allows the identification even of small neuroendocrine tumors, and it suggests an essential tool for the assurance of the diagnosis, and the follow up. The role of the laparoscopic approach and the extent of lymph node dissection in such cases remains controversial, but with this case we suggest the use of laparoscopic approach is feasible, in combination with pre- and postoperative 68-Ga-DOTATATE PET CT imaging.

## Data Availability

The original contributions presented in the study are included in the article/Supplementary Material, further inquiries can be directed to the corresponding author.
